# Wireless intravesical device for real-time bladder pressure measurement: Study of consecutive voiding in awake minipigs

**DOI:** 10.1371/journal.pone.0225821

**Published:** 2019-12-02

**Authors:** Mohammad Ayodhia Soebadi, Marko Bakula, Lukman Hakim, Robert Puers, Dirk De Ridder

**Affiliations:** 1 Laboratory of Experimental Urology, Department of Development and Regeneration, KU Leuven, Belgium; 2 Department of Urology, Faculty of Medicine, Dr Soetomo General Academic Hospital, Universitas Airlangga, Surabaya, Indonesia; 3 Department of Urology, Universitas Airlangga Hospital, Faculty of Medicine, Universitas Airlangga, Surabaya, Indonesia; 4 ESAT-MICAS, KU Leuven, Belgium; University of Houston, UNITED STATES

## Abstract

Traditional urodynamics have poor correlation with urological symptoms. Ambulatory urodynamics may improve this correlation but the need for a transurethral catheter and the time-consuming nature of this examination limits its use. Therefore, the objective of this study was to develop a wireless real-time bladder pressure measurement device for repeated and prolonged-term measurement of bladder behavior in awake pigs. The Bladder Pill is an intravesical device with a pressure microsensor and a 3-dimensional inductive coupling coil for energy supply. A corresponding external coil provides wireless power transmission and real-time communication of bladder pressure data. To test the correlation between the pressure data measured by the device and by standard methods, we compared static water column pressures with this device and water-filled urodynamic catheter systems. In vivo assessment of awake voiding by the pill was done by introducing the bladder pill into the bladder of Göttingen minipigs. An air-charged urodynamic catheter was introduced transurethrally as control for pressure measurements. The optimal physical configuration of the pill was investigated to maximize the containment in the bladder. We used two versions of external signal receivers (one waistband and one rectangular frame) to test the optimal external signal capture. Next to that, we performed short-term and medium-term comparative pressure studies. The in vitro static pressure measurement demonstrated a mean difference of less than 1 cm H_2_O between the methods. The optimal design of the pill for maximal retainment in the bladder proved to be a pigtail configuration. The bending of the device during bladder contractions caused offset of 2.7 +/- 1.4 cm H_2_O (mean +/- SD) on the pressure measurements. The rectangular frame performed signal capture during 5 consecutive voids with a good correlation of the pressure measurements. The device can be inserted through the urethra and is retrieved using string or endoscopic extraction. In conclusion, wireless long-term measurement of bladder pressure is demonstrated and yields comparable results to current available catheter methods of measurement in a pig model.

## Introduction

Bladder pressure is a key parameter measured in the objective assessment of lower urinary tract dysfunction. [1] In neurogenic bladder conditions, abnormal patterns of intravesical pressure are associated with increased risk for upper urinary tract damage and renal dysfunction. [2] In the condition of idiopathic overactive bladder syndrome, findings from urodynamic investigations have poor correlation with reported symptoms. In 36% of patients with symptoms, there are no overactive detrusor contractions and vice-versa in 20–36% of examinations where overactive contractions are measured, patients have no symptoms. [3,4] To improve this correlation, ambulatory urodynamics have been introduced. The longer duration of observation has increased detection of urodynamic abnormalities such as detrusor overactivity. [5,6] However, this technique has seen limited usage in practice. Catheters may cause discomfort or become dislocated. [7] The equipment is cumbersome and the examination requires more time from patients, care/nursing staff and interpreting urologists. [8,9]

The standard recommendation for bladder pressure monitoring is a urethral catheter connected to a fluid-filled external transducer. [1] Other mechanisms are air-filled catheters and microtip sensors, with limitations specific to each catheter type. [10] However, common inconveniences to all catheter types are the limited observation time and subject awareness of the measuring instrument.

Several implantable devices based on pressure microsensors have been proposed as alternative to catheters. [11–15] Key features of these devices are data readout and power supply which both contribute to the device dimensions. Other problems in common with catheters are the insertion methods and intravesical retainment mechanism.

In this study, we use the Bladder Pill, a device with a novel radio frequency induction system for both power and real-time pressure readout. A previous version of this device used battery and memory storage within the device, limiting duration of observation and necessitating extraction and disassembly for data analysis. [11] Recently, an updated design has implemented wireless induction technology and showed promising results in limited in vivo testing. [16] The primary aim of this study was to determine the feasibility and characteristics of pressure measurement using the Bladder Pill in comparison to current catheter systems in vitro and in vivo. The secondary aim was to study techniques to extend the duration of measurements.

## Materials and methods

### Intravesical device and external circuit

**Fig 1:**
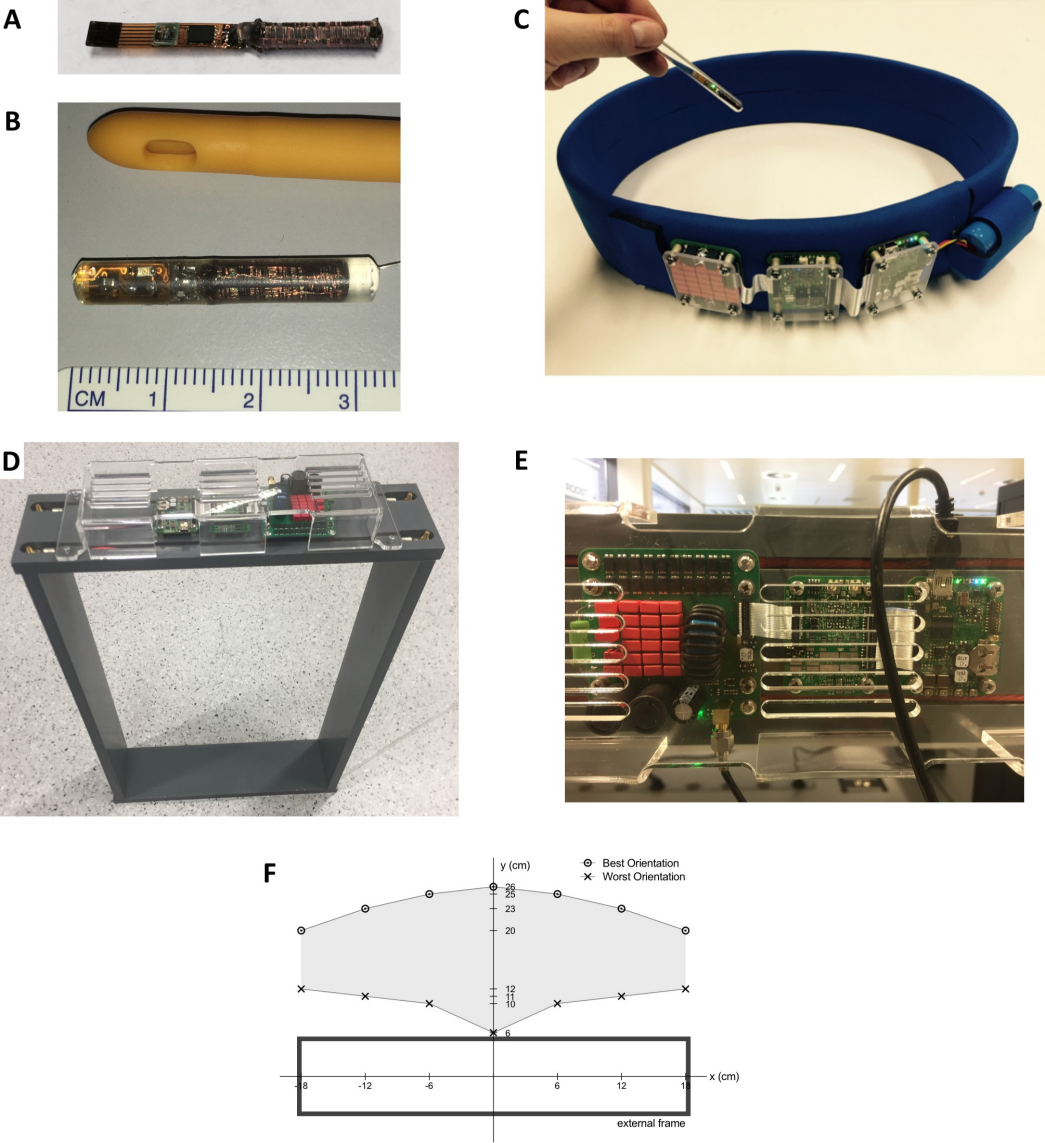
The Bladder Pill. **Device design and components of a wireless intravesical real-time bladder pressure monitoring device.** (A) Non-encapsulated internal components. (B) Encapsulated Bladder Pill alongside an 18 Fr catheter. (C) Pill with the waistband containing external transmitting coil, circuit and battery (Used by permission from Bakula et al, 2016). (D) External frame device. (E) Close-up view of electronics on external frame. (F) Operating range of a Bladder Pill specimen with 5W input power. Operating range was defined as the distance in centimeters where the average sampling rate decreased <1 Hz. Best orientation was the Bladder Pill axis in parallel to the y-axis of the diagram. Worst orientation was pill in parallel to x-axis and rotated until weakest power transfer was attained.

The measurement system consists of the Bladder Pill and a corresponding external device (Fig 1). The pill contains an MS5637 pressure sensor microchip (Measurement Specialties, New Jersey, USA) and a custom 3-dimensional power receiving coil encapsulated in medical-grade silicone tubing (4.6 mm diameter–Hilltop Products Ltd., Kirkstead Way, Golborne, Warrington, UK) and is vacuum-potted with Sylgard 184 silicone elastomer (Dow Corning, USA) Specifications are listed in Table 1 and we refer to previous publications for further technical details. [11,16] The design of the Bladder Pill featured soft encapsulation and flexibility for urethral insertion in humans through a customized 16 Fr urinary catheter. This insertion technique was not yet applicable for this study due to difficulty of urethral access in the animal model.

**Table 1:** Device specifications.

**Implantable device**	
Dimensions	Length 30–40 mm Diameter 4.6 mm
Equivalent French diameter	15 Fr
Weight	1.0 g
Sampling rate	Up to 10 Hz
Pressure range	300–1200 mbar
**External device**	
Waistband circumference	75 cm
Rectangular frame	36 x 55 cm

The external device contains a transmitting coil to energize and to exchange data, as well as all the mandatory power and communication circuits. Pressure and temperature data were transmitted in real-time. Two different designs of this external device were elaborated: a waistband and a free-standing rectangular frame (Fig 1C, 1D, 1E and 1F). The waistband was used for in vitro bench studies. The waistband-formed external device had a sinusoidal coil layout and covered by 3-mm thick neoprene. [16] There was a rechargeable battery and 3 printed circuit boards on the exterior interlinked with ribbon cables.

**Fig 2:**
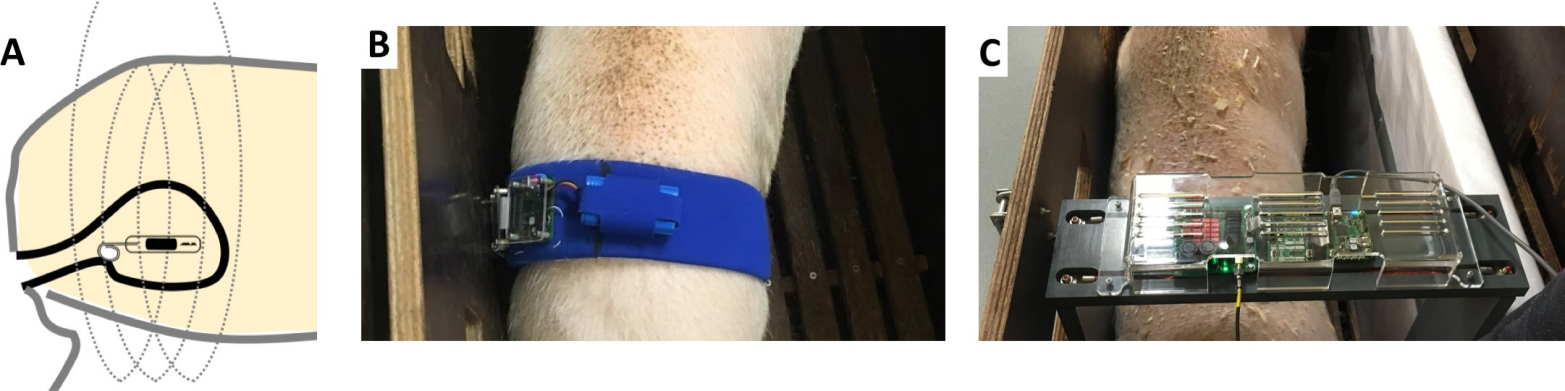
Principle of Bladder Pill for pressure measurement in animals. (A) Diagram of working mechanism of Bladder Pill in vivo, wireless induction as represented by coils (dotted lines) contained within a corresponding external device. (B) Waistband-form external device tested in vivo. (C) Rectangular frame.

Initial animal pilot studies proved that the belt was not well tolerated by the pigs, which led to the design of a rectangular “frame” to avoid direct cutaneous contact (Fig 2). This frame was used to obtain all quantitative data from animal experiments. The inductive powering frame is fabricated from 15mm-thick PVC plastic sheet which encases a single-turn inductive loop. Inside dimensions of the frame were 36 x 55 x 10 cm—width, height and length, respectively. The electronic circuits from the belt device were re-used with an addition of an RF step-up transformer and an external power supply, which replaced the battery to increase the possible output power to over 5W. Functional range of the Bladder Pill was considered as the distance where average sampling rate fell below 1 Hz. As shown in Fig 1F, this distance was more than 20 cm, as measured from the center of the frame in the most optimal orientation of the pill (longest dimension pointing towards the center of the frame). The pill can operate in a wide range of angles anywhere within the frame, except for a small subset of angular "dead zones" where little or no magnetic flux is captured by any of the 3 internal power-receiving coils. This typically occurs when the axis of the pill is near-parallel to the frame face, suggesting that this configuration is close to practical limits of pure inductive powering and communication due to its very weak coupling coefficient. Nevertheless, this caused little interference with the experiments since the position of the pill was fixed by tying it to the catheter. Most interruptions were caused by the animals walking out of range, requiring adjustment of the frame position. On the contrary, the belt-type data logging unit, which remains a much more likely candidate for future use in humans, showed stable reception with no dead zones at a cost of being less well tolerated by the animals.

### Bench testing

**Fig 3:**
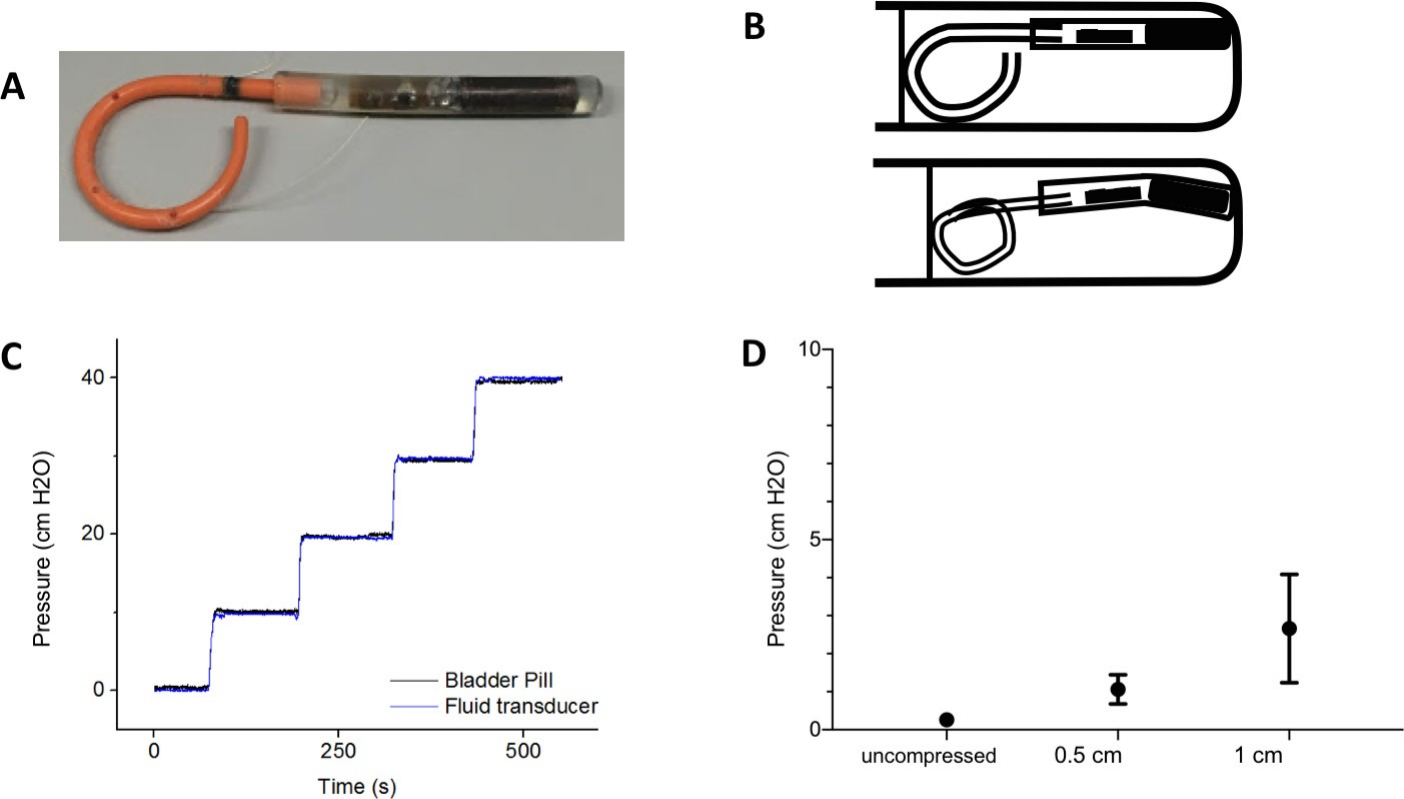
In vitro study of Bladder Pill and fluid-filled pressure measurement catheter. **(**A) Bladder Pill with polyurethane tail attached as retention mechanism. (B) Schematic diagram of longitudinal compression. (C) Simultaneous measurement of pressure generated by static water column between Bladder Pill and fluid-filled pressure transducer. (D) Effect of longitudinal compression on difference in measured pressure between Bladder Pill and fluid-filled pressure transducer. (The Bladder Pill was used in combination with waistband device for these studies).

Linear response of the Bladder Pill device was previously characterized in pressure chambers for values up to 300 cm H_2_O above atmospheric pressure. [11] We compared measurement of static water columns by the Bladder Pill and the waistband external device to a fluid-filled catheter system. A PE-50 polyethylene catheter was connected to a fluid pressure transducer (TSD104A, BIOPAC Systems, Goleta, USA). To simulate the effect of bladder contractions on the Bladder Pill measurements, we inserted the device into a syringe and compressed the Pill longitudinally, as shown in Fig 3. Data presented in this section was obtained using the Pill combined with the waistband external device.

### Experimental animals

Animal experiments were approved by the Ethical Committee for Animal Research of University of Leuven. Animal care and procedures were performed in accordance with national regulations on animal studies and the Guide for the Care and Use of Laboratory Animals (National Institutes of Health). [17] Urethral insertion of devices was performed in five adult female Gottingen minipigs of 24 months age weighing 60–70 kg (Ellegaard Minipig A/S, Dalmose Denmark). To limit artefacts in awake animal measurements, we used TDOC-7FDR three-way air-charged catheters (ACC) connected to a Laborie Aquarius TT urodynamic system (Laborie Medical Technologies Europe, Bristol, UK). [18] Animals went inside a transport cart measuring 60 x 130 x 90 cm during experiments. Anesthesia was necessary for urethral instrumentation with ketamine (15 mg/ml i.m.) and xylazine (2 mg/kg i.m.). The urethra was identified by a combination of palpation and speculum visualization. Access into the urethra and bladder was confirmed by successful insertion of an 8 Fr catheter and urine flow. A guidewire was introduced through the catheter and the catheter was then replaced by a cystoscope sheath. For simultaneous comparison study, the Bladder Pill was secured adjacent to the air-charged catheter balloon sensor and inserted through the sheath lumen. This helped to minimize the effects of hydrostatic pressure offset by keeping the pill and balloon at the same location and also be able to easily retrieve the pill without anesthesia. The external circuit, either the waistband or the frame, was placed around the animal midsection. After recovery from anesthesia marked by spontaneous return to standing, we infused normal saline at body temperature at a rate of 50 ml/minute. This rate was chosen based on medium fill rate and the practice of filling cystometry in children (10% of voided volume/minute). [1,19] Baseline urine collection and filling cystometry of the minipigs used in this study were performed, with voided volumes of 516 +/- 364 ml (n = 34 voids) similar to published values. [20] Filling was continued until voiding was observed by characteristic sustained pressure rise and visual observation of urine flow. Once the measured intravesical pressure in either pill or catheter measurement returned to baseline, infusion was restarted. This was repeated until 5 consecutive voiding events were observed. During pilot urodynamic studies, no residual urine measurement was obtained by disconnection of infusion tubing and spontaneous drainage. Meanwhile, syringe aspiration resulted in occasional inadvertent catheter removal and limited aspired volume possibly due to the small caliber of catheter infusion port of the 3-way catheters. We chose to focus on simultaneous pressure measurement of multiple voids while minimizing the need for additional anesthetic administration in case of catheter dislodgement. The protocol of filling cystometry as performed is available (dx.doi.org/10.17504/protocols.io.3eggjbw). During filling cystometry, we observed the animal continuously and logged relevant events. Time registration of voids was noted when identified from the typical rise in bladder pressure and flow of urine was visually observed.

### Data processing and statistical analysis

Pressure values were reported in cm H_2_O. Results are expressed as means +/- standard deviation unless otherwise specified. To compare the measurement systems, values were corrected for initial deviation from zero baseline as described by Gammie (2016). [1,21] Both catheter and device pressure measurements were filtered using a 0.5 Hz fourth-order low-pass Chebyshev filter based on human physiologic bladder events. [22] We also compared baseline pressure for each filling cycle as measured. This was defined as the minimum pressure between voiding as determined from a smoothed original record (running smoothing average of 50 data points, sampling rate 10 Hz). Data filtering and further processing was performed with Origin Pro 9.0 (OriginLab Corporation, Northampton, MA, USA).

The intraclass correlation coefficient was calculated to quantify the reliability between the measurement methods. [23] Bland-Altman analysis was used to quantify the difference in values measured. Baseline pressures between the two devices were compared, with means compared by unpaired t-test and variances by F-testing. A p value < 0.05 was considered to indicate a statistically significant difference. Statistical analyses were performed using Graphpad Prism 7.0 (GraphPad Software Inc., San Diego, California).

## Results

Design considerations of the Bladder Pill accounted for suitability of later use in humans. The intravesical device is small enough to pass through a cystoscope or to be mounted at the tip of a catheter. Soft and flexible external encapsulation allows later extraction by a string attachment without additional instruments (comparable to indwelling ureteric stents). [24]

In vitro static water column pressures measured by the Bladder Pill in combination with the waistband external device demonstrated agreement with fluid-filled transducers (difference 0.263 +/- 0.154 cm H_2_O, ICC 1.0) and ACC (difference 0.079 +/- 0.076 cm H_2_O, ICC 0.996). Longitudinal compression increased the difference of the Pill measurement compared to fluid-filled catheters by 1.06 +/- 0.38 cm H_2_O at shortening of 0.5 cm and 2.66 +/- 1.42 cm H_2_O at 1 cm (Fig 3).

**Fig 4:**
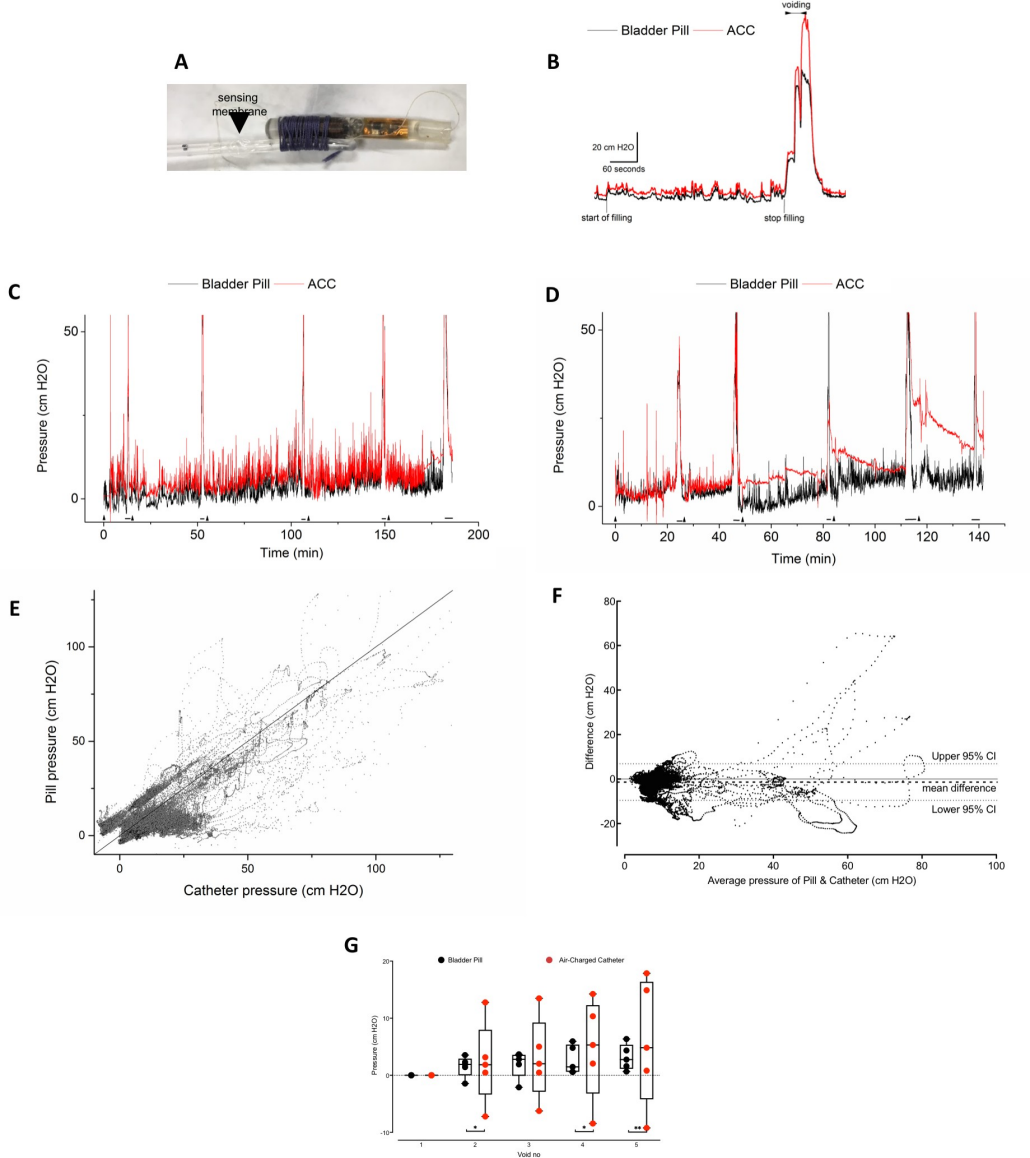
Awake consecutive voiding study in minipigs. (A) Pill tied adjacent to the sensing membrane of the air-charged catheter (ACC). (B) Detail of a single void, events as marked on the graph. (C) & (D) Sample recording 1 & 2, illustrating five consecutive voids in a single measurement session. The baseline drift and the reduced responsiveness of the ACC was observed at the last filling of Fig 4C and starting from third filling of Fig 4D. The ACC unexpectedly registered reduced variation expected from breathing. Arrowheads mark start of filling, and horizontal lines mark voiding events. (E) Correlation plot of pressure at all time periods (Intraclass correlation coefficient r = 0.725, 95% confidence interval 0.723–0.727). Diagonal reference line shows where all points would fall if both measurements agreed perfectly. (F) Bland-Altman plot of differences between pressures measured by Bladder Pill and ACC (bias = -2.7 cm H_2_O, 95% limits of agreement -15.7 to 10.4 cm H_2_O). (G) Change of baseline pressure in each micturition cycle between Bladder Pill and ACC (initial void = 0). Statistical analysis using unpaired t test with Welch’s correction for unequal variance (Void 2 p = 0.85, Void 3 p = 0.78, Void 4 p = 0.63, Void 5 p = 0.61). Larger variance of baseline value changes were observed from the ACC than the Bladder Pill in the second, fourth and fifth voids (F test for variance Void 2 p = 0.023, Void 3 p = 0.054, Void 4 p = 0.034, Void 5 p = 0.010). Data presented in red was obtained from the air-charged catheters (ACC), data in black from the Bladder Pill or a direct comparison of both methods (Fig 4E & 4F).

Initial testing of the system with the waistband on minipigs established a satisfactory wireless induction tuning. However, we encountered several limitations of our animal model. First, the minipigs voided infrequently under anesthesia even with bladder filled to capacity. Second, attempts to restrain the minipigs in a custom hammock was unsuccessful due to excitation and animal struggling during recovery from anesthesia. In contrast, animals with only urethral urodynamic catheters inserted were able to tolerate observation of multiple consecutive filling and voiding cycles. This forced us to design a non-contact rectangular frame within the inside of the animal cage, containing the inductive powering loop along with the same circuits as used in the waistband device. This allowed continuous induction powering and repeated measurement of voiding while the animal is standing freely. With this frame, every animal tolerated 5 consecutive fill-void cycles (duration 119.4 +/- 43.7 minutes, filling volume 900 +/- 360 ml, Fig 4B, 4C & 4D). Corresponding changes in bladder pressures were observed on both the Bladder Pill and the reference catheter measurement (Fig 4, ICC r = 0.727). The pressure measured by both methods, Bladder Pill and the air-charged catheter (ACC), differed by -2.7 +/- 7.9 cm H_2_O (Fig 4F). In comparison of baseline pressures by episodes of consecutive voiding, there were no difference in means of voids 1–5, however, the ACC had larger variance in voids 2, 4 and 5 than the Bladder Pill (Fig 4G).

We explored longer term implantation by attaching a 3 cm section of polyurethane loop from a self-retaining ureteral catheter to the dummy pills as to prevent expulsion (Fig 3A). Pills were retained in the bladder until extraction at 7 days in all 5 animals. Extraction was performed by endoscopy using human instruments in 4 animals. In 1 animal, inadequate length of instruments necessitated open surgery.

## Discussion

Catheters are accepted as the standard in bladder pressure measurement with specific limitations to each catheter technology. Intravesical devices have the potential for less intrusive and longer term bladder pressure monitoring. Overall, the results from this study showed that the Bladder Pill system is feasible for measurements of bladder pressure in an animal model. In conditions of repeated voiding measurements, this system outperforms catheters in quality of data. With an external device in the form of a waistband, this system can potentially measure bladder pressure continuously over a 24 to 48 hour period, avoiding the discomfort of an indwelling urethral catheter.

Telemetry of bladder pressure was previously reported with surgically implanted devices in minipigs [13]. Previous designs of intravesical devices had data storage within the device, therefore measurement data was only available after completion of measurement and successful retrieval. [12,25] Clasbrummel et al have independently proposed a similar wireless system as described in this study. [15] Majerus et al have also reported their work on a pressure sensor for submucosal implantation with wireless battery recharging. [14,26] In this study, we have demonstrated the feasibility of wireless induction in vivo for pressure measurement using a catheterless device in the bladder lumen for both power and real-time data communications. Pressure data were immediately available during the measurement procedure.

The pressures measured in this study were within the expected physiological range of intravesical pressures observed in previous porcine studies. [13,27] We showed, in vitro, the agreement of pressures measured by the Bladder Pill with catheters connected to fluid-filled transducers. However these catheters are susceptible to movement and tube knock artefacts and therefore not suitable for awake animal experiments. [28,29] Therefore, we used the ACC as a comparison pressure method. We decided to compare values from the entire observation period. The detrusor pressure was not calculated because we did not measure abdominal pressure values.

The mean difference between the measurements seen in this study is comparable to other studies of this catheter type. [30,31] Furthermore, pressure values measured by different methods have a wide range. Studies attempting simultaneous pressure measurement in vivo using two instruments in the bladder are limited. Comparison of ACC with fluid-filled catheters in women showed similar spreads of pressure measurement, although this study did not include analysis of cough or void events. [32] In 62 patients recruited for simultaneous comparison of catheters, more than half of the recordings were excluded due to data issues where 12 of these were problems in the voiding phase. [21] In comparative studies of other catheter types, increased difference at higher pressures were also observed but with fewer observation points. [31,33] These comparison studies have has reported a wide spread of values despite small mean differences. Gammie et al. compared ACC with fluid-filled catheters during change in posture, from supine to standing. This study found a mean difference of 0.9 +/- 5.0 cm H2O (mean +/- SD), with 95% limits of agreement of -10.7 to 8.8 cm H2O. [21] A second study comparing the same catheter types concluded that ACC measurements were consistently higher, wide variations in readings were found, but these differences did not change the urodynamic diagnoses in the study population of women. [32] Hundley et al. compared microtip catheters with fluid-filled catheters at cough and Valsalva maneuver and reported differences of at minimal Valsalva 7 +/- 12 cm H2O (mean +/- SD) to 24 +/- 27 cm H2O (mean +/- SD) at maximum cough. [33]

In 4 of 25 filling cycles we observed an unexpected reduction in pressure variation from the ACC (Fig 4C and 4D), while the Bladder Pill had a more stable output. Furthermore, the baseline pressure between micturitions of ACC showed a larger spread of values compared to the Bladder Pill (Fig 4D & 4G). Possible reasons for this variance may originate from a true change in the baseline pressure measured or from drift of the sensing system. The pressure may increase due to residual urine which was not evacuated after each void, although in this case changes in baseline pressure would have equally affected both measurement systems. After micturition, the empty bladder may cause vertical displacement of both measurement systems. The pendulous abdominal wall or shift in other abdominal contents may also account for variation in intraabdominal pressure. Finally, prolonged measurement using the ACC has been reported with average drift of 4% at 2 hours, which corresponds to the median duration of continuous measurement in the current study. [18]

The voiding curve of minipigs had features similar to murine cystometry except for the absence of the oscillatory sphincter activity [34]. At the end of the detrusor contraction, a rise in bladder pressure was observed in our study, similar to previous findings in minipigs [13,27] as well as in a proportion of child [35] and adult cystometry in humans. [36,37] From the scatterplots of measured pressures (Fig 4E & 4F) we observe a wider spread of pressure measurements in higher pressure values. The factors affecting baseline pressures as previously discussed may also contribute to this finding. The high pressures measured may originate external from the bladder (i.e. coughing and straining) as well as from bladder contractions during voiding. We considered the impact of static bending on Bladder Pill measurement characteristics in bench experiments and found a deviation of less than 5 cm H2O, although dynamic transient increases by powerful bladder contraction cannot be excluded (Fig 3). Finally, timestamps of the separate pressure registrations may be slightly misaligned.

During observation of animals in short term and longer indwelling experiments, no change in animal behavior was observed. Any effect of the device on sensation and voiding mechanics remain uncertain. Additional design features can provide buoyancy and device retention mechanisms while minimizing effect on bladder sensation and voiding mechanics.

Further development of this device can address the current limitations. The Bladder Pill used in these experiments were sufficiently small to pass through a cystoscope or to attach at the end of a urethral catheter. The current device utilizes off the shelf components, therefore use of more integrated components in future development may further decrease the dimensions. Technical solutions for current limiting factors of wireless connection and durability are available. Implantable devices employing similar principles are already widely used in capsule endoscopy and temperature measurement, [22,38,39] although use of pressure sensors require extra attention to calibration and drift. [22] Algorithms based on signal properties of bladder contraction and extravesical motion can classify relevant bladder events to substitute for abdominal pressure measurement. [40] In future human studies, the absence of instruments remaining within the urethra and disrupting the sphincter mechanism can contribute to improved urodynamic fidelity and improved comfort.

## Conclusions

We demonstrated feasibility and performance of a small wireless bladder pressure measurement device in vitro and in vivo. Bench testing demonstrated agreement of pressure measurement with catheter-based methods. The effect of longitudinal compression of the pill on pressure measurement is minor, and probably clinically irrelevant. From animal studies, there is no difference in voiding pressures compared to the catheter-based methods. Additionally, the Bladder Pill outperforms the air-charged catheters for measurements of multiple voids and longer duration. Moreover, the Bladder Pill device allows for unobstructed daytime recording of bladder function without hindering the comfort of the patient. This creates a new opportunity in understanding human bladder behavior.

## Supporting information

S1 File ARRIVE checklist. (PDF)

S2 File Operating range data. (TXT)

S3 File In vitro data. (ZIP)

S4 File In vivo data. (ZIP)

S1 Fig Previous version of Fig 1. Original figure from previous publication by the same authors adapted for a previous version of Fig 1.
(JPG)
