# Supplementary figures and images for: Wireless intravesical device for real-time bladder pressure measurement: Study of consecutive voiding in awake minipigs
Source: PLoS One. 2019 Dec 2;14(12):e0225821. doi: 10.1371/journal.pone.0225821 (PMC6886791; doi:10.1371/journal.pone.0225821)

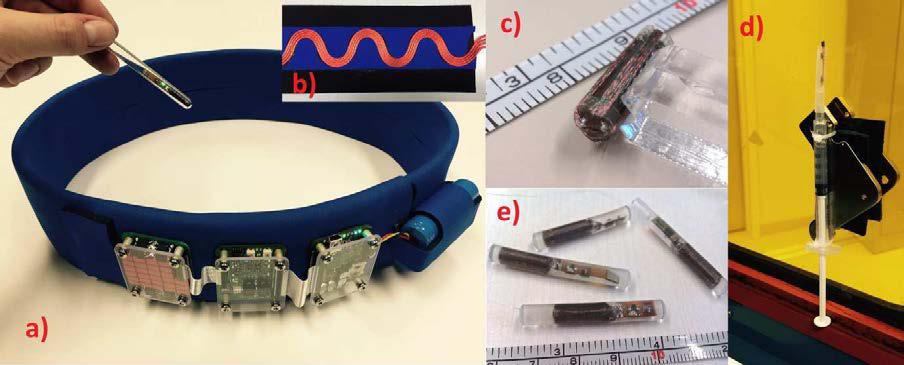

Supplement: S1 Fig — Original figure from previous publication by the same authors adapted for a previous version of Fig 1. (JPG) [file pone.0225821.s005.jpg]
